# Restricted truncal sagittal movements of rapid eye movement behaviour disorder

**DOI:** 10.1038/s41531-022-00292-0

**Published:** 2022-03-15

**Authors:** Danielle Wasserman, Silvia Gullone, Iain Duncan, Mattia Veronese, Valentina Gnoni, Sean Higgins, Adam Birdseye, Emine Cigdem Gelegen, Peter J. Goadsby, Keyoumars Ashkan, K. Ray Chaudhuri, Giulio Tononi, Panagis Drakatos, Ivana Rosenzweig

**Affiliations:** 1 Sleep and Brain Plasticity Centre, Department of Neuroimaging, Institute of Psychiatry, Psychology and Neuroscience (IoPPN), King’s College London (KCL), London, UK; 2 Sleep Disorders Centre, St. Thomas’ and Guy’s Hospital, GSTT NHS, London, UK; 3 Department of Neuroimaging, Institute of Psychiatry, Psychology and Neuroscience (IoPPN), King’s College London, London, UK; 4 Department of Basic and Clinical Neuroscience, Institute of Psychiatry, Psychology and Neuroscience (IoPPN), King’s College London, London, UK; 5 NIHR-Wellcome Trust King’s Clinical Research Facility, King’s College London, London, UK; 6 Department of Neurosurgery, King’s College Hospital, London, UK; 7 King’s College London and Parkinson’s Foundation Centre of Excellence, King’s College Hospital, London, UK; 8 Department of Psychiatry, University of Wisconsin-Madison, Madison, WI USA; 9 Faculty of Life and Sciences Medicine, King’s College London, London, UK

**Keywords:** Parkinson's disease, Neurodegeneration

## Abstract

Unlike sleep-walkers, patients with rapid-eye-movement-behaviour disorder (RBD) rarely leave the bed during the re-enactment of their dreams. RBD movements may be independent of spatial co-ordinates of the ‘outside-world’, and instead rely on (allocentric) brain-generated virtual space-maps, as evident by patients’ limited truncal/axial movements. To confirm this, a semiology analysis of video-polysomnography records of 38 RBD patients was undertaken and paradoxically restricted truncal/thoraco-lumbar movements during complex dream re-enactments demonstrated.

Understanding how dream mentation and ambulation arise from sleep’s basic physiological components has enticing, and potentially wide reaching translational clinical implications^1,2^. A case in point is the rapid eye movement (REM) behaviour disorder (RBD), a relatively rare parasomnia that predicts the later occurrence of alpha-synucleinopathies such as Parkinson disease (PD), multiple system atrophy and dementia with Lewy bodies^3^.

RBD is characterised by the lack of typical REM-like atonia^3^, and by the presence of abnormal behaviours, which can include impressive dream-action isomorphisms^1^, rarely accompanied with significant tachycardia^1,4^. Indeed, the autonomic dysfunction and a reduced phasic and tonic heart rate variability have been demonstrated in RBD patients^4^. Abnormal motor events in RBD range from elementary movements to vivid dream enactments, which preferentially arise from the phasic part of REM sleep^5^ and which present an unique potential to study dreams in an “online” manner^1^.

Previous studies have suggested that dream mentation in RBD may contain more aggression than do the dreams of healthy individuals^1^. Furthermore, this violent trait appears in sharp contrast with the commonly equable disposition of RBD patients during wakefulness^1,6^. Patients with RBD habitually report being attacked or chased, or having to defend themselves or their loved ones during a brief, single visual scene^1,7–9^. This has led some to propose that dreams’ mentation in RBD may be representative of the more rudimentary, evolutionary ‘*fight of flight*’ rehearsal function for the dreams^1,10^. Nonetheless, non-violent elaborate behaviours, such as smoking, singing, gesturing thumbs up, also occur, and they frequently resemble the learned behaviours that are in gross keeping with the cultural and societal norms^1,7–9^.

While commonly described as vivid and engaging, RBD movements almost never involve leaving the bed^11^. Conversely, ambulation in non REM (NREM) parasomnia that can also include a dream re-enactment^12^, suggests an effectual, if incongruous, spatial navigation of the dream environment with patients’ complex procedures ranging from sitting up, leaving the bed, walking down the stairs, cooking or even driving the car^12,13^.

Burgess’ and others seminal work over the last few decades established that successful spatial memory formation and navigation during wakefulness requires striatal reinforcement learning based on *egocentric* representations of sensory states and actions^14^. This sensory information is then incidentally linked with *allocentric* state representations in the hippocampus, with the final adjudication of both outputs subsequently based on confidence/uncertainty in medial prefrontal cortex (for an in-depth review of the field refer to ref. ^14^). Thus, broadly speaking, the brain’s spatial navigation requires landmark coding from two perspectives, relying on viewpoint-invariant (*allocentric*) and self-referenced representations (*egocentric*)^15,16^. Within the *egocentric* neurocircuitry, spatial tuning occurs relative to the self’s body. This is based on the integration of the multiple sensory ascending feedback that allows for the spatial information about the location of the individual in the environment to be formed^16^. Conversely, the *allocentric* representation relies on viewpoint-invariant co-ordinates. For instance, within this reference, spatial inferences are coded based on object-to-object relationships, perhaps not dissimilar to the brain’s own atlas or map of the surroundings, which is formed independent from the individual’s point of view^16^. During spatial navigation, the neural encoding of spatial information occurs within each reference frame but their interactions and functional dependency remain ambiguous^15^.

We are yet to understand if identical neural *egocentric* (e.g., striatal system) and *allocentric* (e.g., hippocampal/primary somatosensory^17,18^) architecture is utilised during spatial exploration of the ambulatory dreamscapes. However, based on our^19^ and others^20,21^ recent work suggestive of early caudate/striatal^21^ and phasic REM changes in RBD patients, we speculate that limited ambulation in RBD reflects an aberrant contribution of the *egocentric* neural architecture during dream mentation. For example, parkinsonisms are known to disappear during RBD‐associated complex movements, suggesting that the upper motor stream bypasses the basal ganglia during REM sleep^22^.

Moreover, utilising a philosophical allegory of people in Plato’s Cave (Supplement, Supplementary Fig. 1), we hypothesise that spatial perception and spatial co-ordinates of RBD ambulation may, unlike NREM ambulation, remain predominantly guided by the (*allocentric*) brain-generated virtual space-maps^17,23,18,24^. Thus, we also demise that no, or very limited, truncal/lumbar axial movement will occur during RBD events across the *yz*-octant of the sagittal plane of the sleeping body (Fig. 1a).

**Fig. 1:**
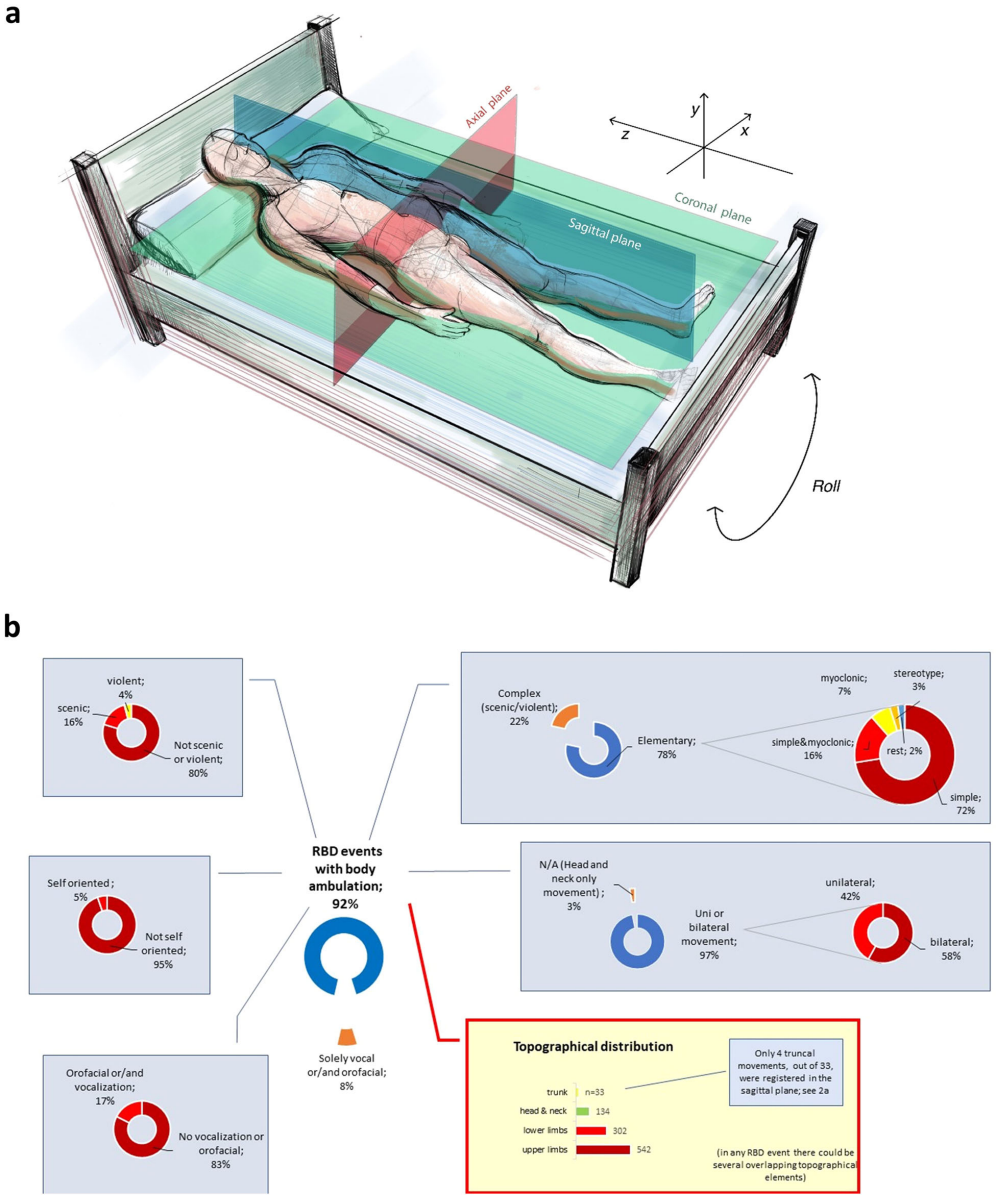
Schematic presentation of the classification of the REM-associated motor movements and spatial planes distribution of truncal movements. **a** Movements were identified in 675 RBD events, while the remaining 62 were vocalisations or/and orofacial events. Out of 675 analysed movements only four truncal (0.59%) movements were registered across the sagittal plane, perhaps suggestive of predominant allocentric spatial dream navigation (*see text*). The semiology analysis of REM-associated motor movements (**b**) is also shown. They were predominantly elementary (*n* = 531), and classified as simple (72.50%). Dream re-enactment was demonstrated in 20.29% of events; upper (80.30%) and lower limbs (44.74%) were the most frequently involved (orange colour indicates patients that were not included into further categorisation. In elementary movements, the ‘rest’ represent one event with both myoclonic and stereotype characteristics, and another one with a mixture of all elementary types. Topographical distribution is visualised via bars since overlapping between several body parts was extensive and did not allow for meaningful grouping as in elementary movements).

In order to fully explore this notion, we undertook a detailed retrospective semiology analysis of video-polysomnography (VPSG) records of 38 newly diagnosed RBD patients (86.84% male; mean age 68.34 (6.98) years) (please refer to Methods; Supplementary Tables 1 and 2).

Seven-hundred thirty-seven discrete RBD events were registered, and 1011 distinct movements of variable duration (mean duration 15.04 (22.23) seconds) were subsequently described and classified (Fig. 1b). Other relevant demographics and polysomnography data are respectively summarised in Supplement’s Supplementary Table 1.

Wide-ranging motor movements were noted in 675 RBD events, while vocalisation was the sole manifestation in 62 RBD events. Limb movements were predominantly noted as fast, repetitive, and jerky. As predicted, the semiology analysis suggested that a truncal change of body plane was absent in 95.12% of registered RBD movements, and movements that led to patient’s sitting on the bed were exceptionally rare. No records of patients vacating their bed, or purposely walking around the room were found.

More specifically, truncal (lumbar spine) body movements formed part of the movements in only 4.88% of all registered RBD events (33 out of 675), which was significantly lower compared to recorded movements of head and neck (19.8%), lower limbs (44.74%), and upper limbs (80.29%) (*x*^2^ (3, *N* = 1011) = 587.5, *P* < .0001) (Fig. 1b). Altogether twenty-nine truncal movements were registered across the axial plane (e.g. *xy*-octants), which predominantly corresponded to the rolling movements of the sleeping body. Notably, only four truncal/thoraco-lumbar movements (0.59%) were registered across the *yz*-octant of the sagittal plane of the sleeping body (*P* < .0001) (Fig. 1a).

To date, only a handful of RBD semiology studies are published, and there is a dearth of VPSG-based RBD-related data^21,25–27^. The additional explicit relevance of our VPSG study is that here, for the first time, primary focus is on categorisation and demonstration of the paradoxically restricted semiology of truncal/thoraco-lumbar movements during RBD events, which persists even during complex dream re-enactments^23^. This notion of a truncally-immobilised avatar body that exists in the *allocentric* dreamscapes (see Supplement, Supplementary Fig. 1) is further supported by findings of a previous study that demonstrated decreased REM-related phasic electromyography (EMG) activity in the thoraco-lumbar paraspinal muscles^28^ in RBD patients.

Increasing body of work suggests that hippocampal plastic changes and neural reactivations during sleep, which underlie encoding and consolidation of long-term memories, are under a distinct homoeostatic control by subcortical neuromodulatory structures and the ponto-geniculate waves.^29^ While their role in modulation of the activity in the egocentric caudate/striatal architecture remains conjectural, it is possible that they dictate transient differential coherence with hippocampal and other wider thalamo-cortical regions^30^. This phasic brain-state co-ordination is yet to be authoritatively demonstrated in the human brain. However, work in primates and other animal models suggests that similar events may lead to attentional shifts that ‘reset’ mnemonic processing frames, as well as result in dream-related conscious experiences^31^. Arguably, these global phasic events may also lead to transitions between discrete epochs of visual-like processing during REM sleep^29^. Early striatal dopaminergic/cholinergic imbalance in RBD patients may also account for specific dream mentation commonly consisting of threatening events^1,21^ (see Fig. 1b).

How specific phasic REM driven neural replay of striato-limbic systems may contribute to a distinct semiology of RBD events registered in our study (Fig. 1), is, however, not entirely clear. Possible alternative explanation may be inferred from two recent discoveries that suggest the primary somatosensory cortex may present a novel somatosensory spatial navigation system outside the hippocampal formation^17,23^. Firstly, intriguingly, spatially selective cells (e.g. place, grid, boundary vector/border and head direction cells) were demonstrated in the rodent’s primary somatosensory cortex^17^. Secondly, this region was shown to exert sensory control over thoracal/lumbar locomotor network, independent of the motor cortex and other supraspinal locomotor centres^23^. Taken together, these findings, along with ours, raise the possibility that paradoxical reductions in truncal movements during RBD events may, at least in part, originate from phasic REM replays across primary somatosensory cortex.

This unexpected shift of locus of interest to the spatial navigation in the primary somatosensory cortex broadly resonates with Brecht’s body avatar model, or model of animatable puppet^18^. Brecht elegantly argues that a neural replay in layer four of primary somatosensory cortex can lead to simulations of the ambulations of sensory body avatar^5^. According to this model, somatosensory superficial layers provide context, as well as storage of sensory memories, and layer-6-to-layer-4 inputs initiate body simulations allowing rehearsal and risk assessment of difficult actions, such as defensive actions or jumps^18^.

Our group recently argued that early synucleinopathy process may lead to changes in the *egocentric* neural architecture (e.g., caudate)^19,21,32^, and consequently, to decreased suppression of frontal beta rhythms during phasic REM^20^. Consecutively, this may also lead to heightened cortical arousal in the primary sensorymotor cortex^20^.

Moreover, any such heightened cortical arousal in patients with RBD could further reduce modular slow-wave activity (SWA) that occurs during phasic REM in layers 3 and 4 of somatosensory cortex^24^. This could in turn result in a reduced sensory disconnection during REM in the primary somatosensory cortex. Notably, any such reduced SWA could also contribute to the increased local cortical activity and the emergence of behavioural episodes^24^, additionally defined by pronounced inhibition of thoracal/lumbar locomotor network^23^.

Arguably, thus, the phasic REM-related replay in the primary somatosensory spatial navigation system may account for most features of RBD events in our patients. Most notably, it might underlie the limited truncal mobilisation during RBD events, and as such it may present a useful biomarker that differentiates the RBD events from other (e.g., NREM) ambulations during sleep. Future multimodal imaging studies should account for the veracity of the proposed distinct activity of the *allocentric* and *egocentric* circuitries during different parasomnia events.

While it is tempting to draw this hypothetic mechanistic inference further, several limitations of our study merit note. For example, due to an observational, and non-controlled nature of our study, no causation or directionality of any RBD semiology relationship can be concurred. Also, due to a retrospective nature and other specifics of the video-analysis-based methodological approach, involving a tertiary centre recruitment-bias, as well as high inter-night variability of RBD^33^, it is possible that elementary, small-amplitude and brief duration motor events were undercounted for in this study. Finally, whether any such extrahippocampal phasic REM replay is independent of, parallel with, or convergent onto, the classical one within the hippocampal-entorhinal microcircuit remains an interesting question that needs to be addressed in future studies.

## Methods

A retrospective analysis of clinical and video polysomnographic (VPSG) findings of all patients diagnosed with iRBD in 2019 based on the American Academy of Sleep Medicine (AASM) classification, at a large tertiary sleep centre was conducted (Supplement, Supplementary Table 1). The study was granted ethical approval by the Hospital Clinic Research Ethics Committee (Project-No-9585, GSTT NHS, UK); according to the strict national guidelines, the study did not require written informed patients’ consents due to use of retrospectively ascertained anonymized data and due to the fulfilment of the following non-negotiable stipulations (1) the study protocol abided by the strictest patients’ data confidentiality and (2) it complied with all requirements of EU General Data Protection Regulation and with the Declaration of Helsinki regulations. Out of forty eligible records, two were excluded, one due to co-morbid narcolepsy type 1 (*n* = 1) and second due to incomplete data recorded in the database (*n* = 1). Thirty-eight patients, of whom thirty six were with no neurologic comorbidity, and two of whom later developed PD (*n* = 1), and unclassified extra-pyramidal symptoms (*n* = 1), respectively, were identified, and their eligibility confirmed through clinical records and the sleep diaries. The semiology of each RBD-event was visually analysed and entered into a statistical database. RBD events were classified into distinct groups according to the predominant motor manifestation (Supplementary Table 2); type of movement, anatomic distribution, synchrony, symmetry, onset, offset, vocalisation, were further evaluated and classified according to the predefined classification, and as previously described (see Fig. 1; Supplementary Table 2). Only motor events with a minimum duration of two seconds were selected and added to the database. Overlapping or successive motor events were scored as a single RBD event, and all motor components were marked. Movements associated with respiratory events or EEG arousals were disregarded. The categorisation of RBD motor events was undertaken, as previously published and further adapted^21,34^. The type of movement, anatomic distribution, synchrony, symmetry, onset, offset, vocalisation, were further evaluated and classified according to the predefined classification (Supplementary Table 2)^34–36^. More specifically, as demonstrated in Table 2, the validated classification according to Frauscher et al.^34^ was used and expanded by parameters utilised in classifications by Terzaghi et al.^35^ and Seneviratne et al.^36^. The video analyses were done independently by the two experienced raters and time of *onset* and *offset* of movements were reported in the database and their *duration* (s) was computed. Only motor events with a minimum duration of two seconds were selected and added to the database. Overlapping or successive motor events were scored as a single RBD event and all motor components were marked. Movements associated with respiratory events or EEG arousals were disregarded.

### Polysomnography acquisition

All subjects had undergone overnight PSG, and the subsequent sleep scoring and video-analysis were performed in accordance with AASM by two experienced sleep technologists. VPSG recordings were obtained using a digital polygraph (*PSG Embla N7000, Natus*) and displayed on *Embla RemLogic* software. The PSG comprised multiple channels: electroencephalogram (EEG, six channels), electrooculogram (EOG, two channels), chin, tibialis anterior electromyogram and in suspected clinical history patients’ additional flexor digitorum was used (EMG), electrocardiogram (ECG), pulse oximeter, thoracic and abdominal respiratory bands, position sensor, nasal flow cannula and infrared video recording. EEG electrodes were placed on the participant following the International 10–20 System^37^. Subsequently, pre-agreed general measures of sleep efficiency were collected, such as total-sleep-time (TST), wake-time-after-sleep-onset (WASO) and sleep latency (SL). Further measures derived from polysomnography that reflect quality and architecture of sleep, included REM latency (e.g., REM L), percentage of total sleep time in NREM stages one to three and REM sleep (e.g., %N1, %N2, %N3, %REM), and those that likely reflected sleep fragmentation, such as periodic limb movement index (PLMI) and apnoea/hypopnoea indices (AHI), were collected (Supplement, Supplementary Table 1).

Scoring sleep stages was done according to standard criteria of the AASM Scoring Manual (*Version 2.4, 2017*), and as previously reported^19^. The selected PSG videos and reports were carefully analysed by two investigators for the purpose of this study. The categorisation of all visible motor events occurring during REM sleep and observed through VPSG was undertaken, please refer to the Supplement and Supplementary Table 2.

### Statistical analyses

Statistical analysis was conducted with Statistical Package for the Social Sciences (SPSS) Statistics 25 (*IBM Corp., USA*). Descriptive statistics for continuous variables included mean and standard deviation. Descriptive statistics for nominal variables was reported using frequencies and percentages. A *chi-square goodness of fit* was calculated comparing the occurrence of each of the four topographical distributions of movements (*N* = 1011 in total, Fig. 1b), with the hypothesised occurrence (25%). A binomial exact test was calculated comparing the occurrence of the two topographical truncal movements, with the hypothesised occurrence (50%). Significance value was set at *P* < 0.05.

Further detailed methodological description of experimental and study procedures, and all the pertinent methodological references, are available in the Supplement.

### Reporting summary

Further information on research design is available in the Nature Research Reporting Summary linked to this article.

## Supplementary information

Reporting Summary

Supplement

## Data Availability

All data that support the findings of this study are available upon reasonable request from the corresponding author.
