# Supplementary material for: Restricted truncal sagittal movements of rapid eye movement behaviour disorder
Source: NPJ Parkinsons Dis. 2022 Mar 15;8:26. doi: 10.1038/s41531-022-00292-0 (PMC8924261; doi:10.1038/s41531-022-00292-0)
Supplement: Supplementary file 2 — Supplement [file 41531_2022_292_MOESM2_ESM.pdf]

Supplement to: Restricted Truncal Sagittal Movements of Rapid Eye Movement Behaviour Disorder

Outline

1 Demographics and Polysomnography Data

- Supplementary Table 1. Demographics and polysomnography data.

2 Semiology of RBD Events

- Supplementary Figure 1. RBD dreaming and allegory of Plato's Cave.

- Supplementary Table 2 – Clinical profile of semiology and motor patterns of RBD events in the patient series.

References

1 Demographics and Polysomnography Data

Supplementary Table 1. Sociodemographic and Polysomnography Data of RBD patients.

		RBD N (%)
**Gender**	Male	33 (86.84%)
	Female	5 (13.16%)
**Medication:** antidepressant	No	31 (81.58%)
	Yes	7 (18.42%)

	Mean	SD	Minimum	Maximum	25th	50th (Median)	75th
**Age** (years)	68.34	6.979	56	85	62	69	74
**BMI** m^2/kg	26.232	3.0737	20.03	31.9	24	27	27.8
**TST**	318.218	58.4916	168.5	414	281.125	326.25	366.65
**WASO** (min)	118.184	66.75.91	17	278.1	60.75	110.150	156.9
**SL** (min)	32.879	60.043	2.8	370	9.375	17.25	30.65
**SE (%)**	66.981	18.2515	0.8	90.7	57.675	68.25	80.25
**REM Latency** (min)	162.224	120.1486	48.5	405.5	69.875	96	262.5
**AHI** (events/hr)	10.829	14.0936	0	54	1.575	5.55	13.5
**PLMI** (events/hr)	29.938	39.8584	0	144.9	3.2	12.1	46.6
**AI** (events/hr)	26.159	13.875	6.8	72.9	16.95	22.9	30.85
**%N1**	21.397	10.9535	3.9	55	13.5	19.2	28.075
**%N2**	39.418	8.4922	25.5	74.1	35.05	37.3	43.05
**%N3**	20.021	9.7465	0.8	40.1	13.72	19.35	26.9
**%REM**	19.032	6.072	4.1	30.6	14.97	20.05	23.475
**RBD event duration** (s)	15.04	22.237	2	320	5	9	16.5

Abbreviations: **%,** percentage; **AI**, arousal index; **AHI**, apnoea/hypopnoea index; **BMI**, body mass index; **N**, number; **N1-3**, non REM sleep stages one to three; **NREM**, non rapid eye movement sleep; **PLMI**, periodic limb movement index; **REM**, rapid eye movement; **SE**, sleep efficiency; **SL**, sleep latency; **TST**, total sleep time; **WASO**, wakefulness after sleep onset.

2 Semiology of RBD Events

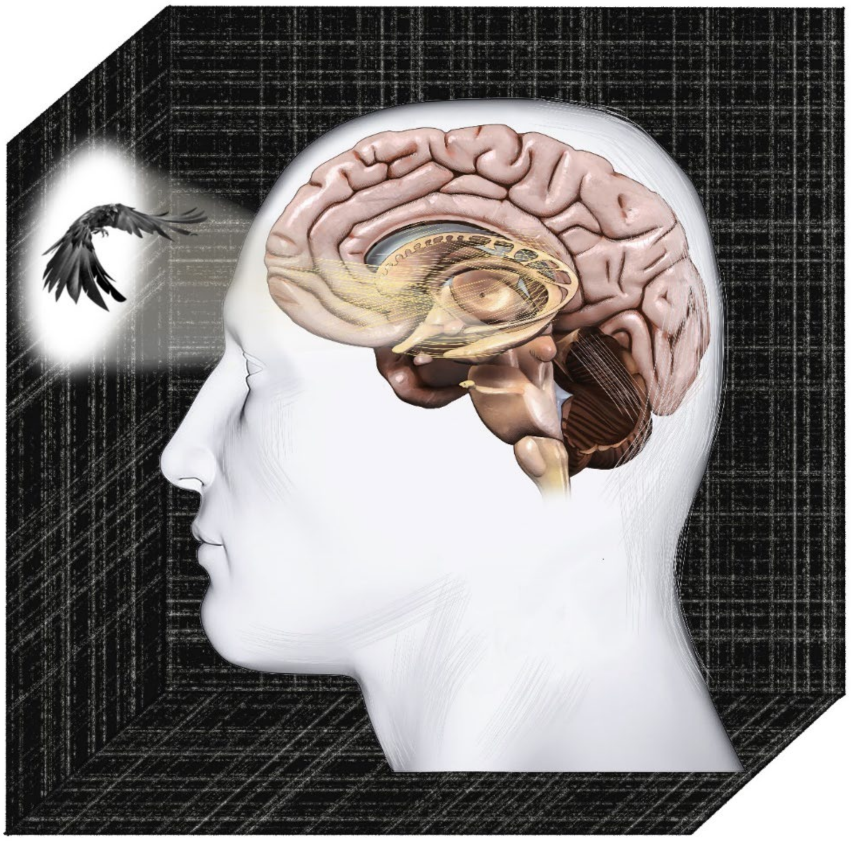

Supplementary Figure 1. **Plato's Cave of RBD dreams**. In the philosophical allegory of Plato's Cave[1], prisoners chained together in a cave fail to identify that the shadows on the wall in front of them are projections of puppets that other people behind them are moving in front of a fire. In a similar manner, patients with RBD, during re-enactment of their dreams, move/ambulate according to the (*allocentric*) brain-generated virtual space-maps[2, 3 4, 5] , oblivious to the spatial co-ordinates of the 'outside world' or their room. Patients with RBD rarely leave the bed, unless this results from their defensive 'jumps or sudden jerky moves' arising from their dream narrative. Thus, commonly no, or a very limited, truncal/lumbar axial ambulation occurs during RBD events (see the main text and **Figure 1**).

Burgess and others established that successful spatial memory formation and navigation during wakefulness requires striatal reinforcement learning based on *egocentric* representations of sensory states and actions[6]. This sensory information is then incidentally linked with *allocentric* state representations in the hippocampus, with the final adjudication of both outputs subsequently based on confidence/uncertainty in medial prefrontal cortex (for an in-depth review of the field refer to[6]). We are yet to understand if identical neural *egocentric* (e.g. striatal system) and *allocentric* (e.g. hippocampal/primary somatosensory[2, 4]) architecture is utilised during spatial utilised during spatial exploration of the ambulatory dreamscapes. In keeping

with a philosophical allegory of people in Plato's Cave, Brecht's Body Model Theory of Somatosensory Cortex similarly argues that a neural replay in layer four of primary somatosensory cortex can lead to simulations of the ambulations of sensory body avatar[7], with somatosensory superficial layers providing context, as well as storage of sensory memories, and layer-6-to-layer-4 inputs initiation of body simulations that allow rehearsal and risk assessment of difficult actions, such as defensive actions or jumps[4].

Supplementary Table 2. Clinical profile of semiology and motor patterns of RBD events in the patient series (n=38).

Type of Movement		Semiology Description *(method adapted from[8, 9, 10])*
Myoclonic events		sudden, brief, jerky, involuntary movements involving extremities, face and trunk[11] (Fahn et al., 1986).
Simple motor events		small-amplitude movements (commonly unnoticeable by a bed partner).
Stereotypes		repetitive, invariant behaviour patterns with no obvious goal or function; automatism-like movements (e.g. smacking, fumbling).
Self-oriented movements		comprised of touching or rubbing one's body part.
Complex events	Scenic	*Complex events* were subdivided into *scenic events,* which described apparent "acting out" of dreams and *violent events,* which referred to large-amplitude, potentially injurious movements (e.g. kicking, punching).
	Violent	
Vocalizations		RBD-related .
Orofacial events		e.g. clenching, grimacing.
Topographical distribution	Head/neck	Distribution of the motor events was established depending on involved body
	Trunk	

	Upper limbs		part(s). Movements were topographically categorized into *head/neck*, *trunk* (rolling and pelvic movements), *upper limbs*, *lower limbs*.
	Lower limbs		
Spatial distribution	Focal	Unilateral	Spatial distribution was subcategorized into *focal* (localized to one body part), *multifocal* (involving non-contiguous body parts), *proximal* and *distal*. *Laterality* of movements was described as *unilateral* or *bilateral*.
	Multifocal		
	Proximal	Bilateral	
	Distal		
Sagittal plane change			the movements that involved truncal/lumbar axial muscles across the yz-octant of the sagittal plane of the sleeping body (see main text Figure 1).

Body movement was identified in 675 RBD events and the remaining 62 were solely associated with vocalisation or/and orofacial events. 531 RBD episodes were not complex and were subcategorised in the following elementary types: myoclonic=35 (6.59%), simple=385 (72.50%), stereotype=14 (2.63%), simple & myoclonic=85 (16.00%), simple & stereotype=10 (1.88%), myoclonic & stereotype=1 (0.18%) and mixture of all types=1 (0.18%). 36 out of the 675 body movement events (5.3%) were self-oriented, scenic=110 (16.29%), violent=27 (4%), vocalisations=29 (4.29%), orofacial=66 (9.77%) and 23 (3.40%) had both. Moreover, the topographical distribution was categorised as follows: head and neck=134 (19.85%), upper limbs=542 (80.30%), lower limbs=302 (44.74%), and trunk=33 (4.88%); and in terms of spatial distribution: focal=211 (35.41%), multifocal=269 (40.59%), proximal=456 (67.70%) and distal=482 (71.7%).Where head and neck were not the only body parts involved (21/675) bilateral body movement was present in 380 (56.29%) of the events.

Traditionally, dream-enactment behaviours in RBD are considered rare[12, 13, 14, 15], commonly occurring in less than 2% of the total RBD events[10, 16]. Conversely, we reported dream re-enactment in 16.29% of recorded RBD events. The percentage of violent behaviours in our cohort, on the other hand, echoed the findings by Frauscher and colleagues in 2007 (3.6%), who included idiopathic RBD cases[10]. Similarly, in accordance to previous RBD studies[10, 12, 17], we found a clear predominance of elementary movements involving upper limbs. In contrast

to these studies, however, we observed a higher number of multifocal, proximal and bilateral motor events with respect to focal, distal and unilateral movements (main text; **Figure 1b**).

References

1. Dale H. INTERPRETING PLATO'S CAVE AS AN ALLEGORY OF THE HUMAN CONDITION. *Journal for Ancient Philosophy and Science* **14**, 74-86 (1980).

2. Long X, Zhang SJ. A novel somatosensory spatial navigation system outside the hippocampal formation. *Cell Res*, (2021).

3. Karadimas SK*, et al.* Sensory cortical control of movement. *Nat Neurosci* **23**, 75-84 (2020).

4. Brecht M. The Body Model Theory of Somatosensory Cortex. *Neuron* **94**, 985-992 (2017).

5. Funk CM, Honjoh S, Rodriguez AV, Cirelli C, Tononi G. Local Slow Waves in Superficial Layers of Primary Cortical Areas during REM Sleep. *Curr Biol* **26**, 396-403 (2016).

6. Chersi F, Burgess N. The Cognitive Architecture of Spatial Navigation: Hippocampal and Striatal Contributions. *Neuron* **88**, 64-77 (2015).

7. Neikrug AB, Ancoli-Israel S. Diagnostic tools for REM sleep behavior disorder. *Sleep Med Rev* **16**, 415-429 (2012).

8. Seneviratne U, Reutens D, D'Souza W. Stereotypy of psychogenic nonepileptic seizures: insights from video-EEG monitoring. *Epilepsia* **51**, 1159-1168 (2010).

9. Terzaghi M*, et al.* Sleep-related minor motor events in nocturnal frontal lobe epilepsy. *Epilepsia* **48**, 335-341 (2007).

10. Frauscher B*, et al.* Video analysis of motor events in REM sleep behavior disorder. *Mov Disord* **22**, 1464-1470 (2007).

11. Fahn S, Marsden CD, Van Woert MH. Definition and classification of myoclonus. *Adv Neurol* **43**, 1-5 (1986).

12. Bugalho P, Lampreia T, Miguel R, Mendonca M, Caetano A, Barbosa R. Characterization of motor events in REM sleep behavior disorder. *J Neural Transm (Vienna)* **124**, 1183-1186 (2017).

13. Manni R, Terzaghi M, Glorioso M. Motor-behavioral episodes in REM sleep behavior disorder and phasic events during REM sleep. *Sleep* **32**, 241-245 (2009).

14. Cygan F, Oudiette D, Leclair-Visonneau L, Leu-Semenescu S, Arnulf I. Night-to-night variability of muscle tone, movements, and vocalizations in patients with REM sleep behavior disorder. *J Clin Sleep Med* **6**, 551-555 (2010).

15. Nguyen-Michel VH*, et al.* Rapid eye movement sleep behavior disorder or epileptic seizure during sleep? A video analysis of motor events. *Seizure* **58**, 1-5 (2018).

16. Arnulf I. REM sleep behavior disorder: motor manifestations and pathophysiology. *Mov Disord* **27**, 677-689 (2012).

17. De Cock VC*, et al.* Restoration of normal motor control in Parkinson's disease during REM sleep. *Brain* **130**, 450-456 (2007).
